# Metabolites derived from bacterial isolates of the human skin microbiome inhibit *Staphylococcus aureus* biofilm formation

**DOI:** 10.1128/spectrum.01306-25

**Published:** 2025-08-05

**Authors:** Viet Hoang Le, Tetyana King, Breanna Wuerzberger, Olivia R. Bauer, Megan N. Carver, Tiffany S. Chan, Annabeth L. Henson, Grace K. Hubbard, Tamar Kopadze, Claire F. Patterson, Sabrina M. McGraw, Aidan O'Hara, Eryk J. Yarkosky, Michael G. LaMontagne, Eileen M. Hotze, Rosana B. R. Ferreira

**Affiliations:** 1 Department of Molecular Biosciences, The University of Kansas 4202, Lawrence, Kansas, USA; 2 Department of Biology and Biotechnology, University of Houston—Clear Lake 14744, Houston, Texas, USA; 3 Department of Undergraduate Biology, The University of Kansas 4202, Lawrence, Kansas, USA; 4 Instituto de Microbiologia Paulo de Góes, Universidade Federal do Rio de Janeiro 28125, Rio de Janeiro, Brazil; Cleveland Clinic Lerner Research Institute, Cleveland, Ohio, USA

**Keywords:** skin microbiota, anti-biofilm activity, *Staphylococcus*, *Bacillus*, biofilms, MALDI-TOF

## Abstract

The human skin microbiome is a diverse ecosystem that can help prevent infections by producing biomolecules and peptides that inhibit growth and virulence of bacterial pathogens. *Staphylococcus aureus* is a major human pathogen responsible for diseases that range from acute skin and soft tissue infections to life-threatening septicemia. Its ability to form biofilms is a key virulence factor contributing to its success as a pathogen as well as to its increased antimicrobial resistance. Here, we investigated the ability of bacterial skin commensals to produce molecules that inhibit *S. aureus* biofilm formation. Matrix-assisted laser desorption ionization-time of flight mass spectrometry (MALDI-TOF MS) identified 77 human skin microbiome bacterial isolates from *Staphylococcus* and *Bacillus* genera. Metabolites from cell-free concentrated media (CFCM) from 26 representative isolates were evaluated for their ability to inhibit biofilm formation by both methicillin-resistant (MRSA) and methicillin-sensitive (MSSA) *S. aureus* strains. CFCM, derived from most of the isolates, inhibited biofilm formation to varying extents but did not inhibit planktonic growth of *S. aureus*. Size fractionation of the CFCM of three *S*. *epidermidis* isolates indicated that they produce different bioactive molecules. Cluster analysis, based on either MALDI-TOF mass spectra or whole-genome sequencing draft genomes, did not show clear clusters associated with levels of biofilm inhibition among *S. epidermidis* strains. Finally, similar biosynthetic gene clusters were detected in all *S. epidermidis* strains analyzed. These findings indicate that several bacterial constituents of the human skin microbiome display antibiofilm *in vitro* activity, warranting further investigation on their potential as novel therapeutic agents.

**IMPORTANCE:**

The skin is constantly exposed to the environment and consequently to numerous pathogens. The bacterial community that colonizes healthy skin is thought to play an important role in protecting us against infections. *S. aureus* is a leading cause of death worldwide and is frequently involved in several types of infections, including skin and soft tissue infections. Its ability to adhere to surfaces and produce biofilms is considered an important virulence factor. Here, we analyzed the activity of different species of bacteria isolated from healthy skin on *S. aureus* biofilm formation. We found that some species of *Staphylococcus* and *Bacillus* can reduce *S. aureus* biofilm formation, although a generally lower level of inhibitory activity was observed compared to *S. epidermidis* isolates. Among *S. epidermidis* isolates, strength of activity was dependent on the strain. Our data highlight the importance of mining the skin microbiome for isolates that could help combat skin pathogens.

## INTRODUCTION

The skin is the primary physical barrier to the human body. This dry and impermeable environment limits bacterial growth through low pH and secretion of antimicrobial molecules (1). Despite this, a multitude of microbes persist in this environment, creating a complex and diverse ecosystem termed the skin microbiome. In healthy individuals, the skin microbiome includes bacteria from the genera *Staphylococcus*, *Cutibacterium*, *Corynebacterium*, *Streptococcus*, and *Micrococcus* (2). Species composition on the skin of an individual host varies between moist, dry, and sebaceous sites (2). Interactions between members of this ecosystem and host factors likely influence the diversity of the skin microbiome and consequently its function.

Interactions between members of the skin microbiome and pathogens can play a role in colonization resistance, aiding in host protection against infections (3). Much of what is known about the mechanisms of colonization resistance has been gleaned from studies of the gut microbiome. Specific mechanisms behind this phenomenon on the skin remain relatively unexplored (4). In general, studies have shown that colonization resistance involves direct and indirect mechanisms, including the production of antimicrobials, nutrient competition, interference with quorum sensing, and stimulation of the host’s immune system. For example, antimicrobial peptides (AMPs), produced by coagulase-negative *Staphylococcus* isolates from the skin microbiome, can inhibit *S. aureus* growth and act synergistically with the human AMP LL-37 (4). In addition, *Staphylococcus epidermidis*, a common member of the skin microbiome, expresses phenol-soluble modulins capable of killing both *Streptococcus pyogenes* and *S. aureus* (5). Furthermore, colonization resistance may occur through indirect methods involving triggering the host immune response (3, 4). For example, *S. epidermidis* modulates the innate immune system by upregulating the production of perforin-2 activating gamma delta T cells in epithelial tissues, which facilitates elimination of intracellular *S. aureus* (6).

Although *S. aureus* is part of the normal human skin microbiome in approximately 30% of the population (2), it is also a major pathogen responsible for human diseases ranging from uncomplicated skin and soft tissue infections to life-threatening pneumonia and septicemia (7). In addition, *S. aureus* accounts for an estimated 20% of surgical site infections (8). The high rate of antimicrobial resistance among *S. aureus* clinical isolates, such as methicillin-resistant *S. aureus* (MRSA), limits the clinical effectiveness of our current therapeutics, leading to higher mortality and morbidity rates and increasing healthcare-associated costs (9). *S. aureus’* success as a pathogen can be attributed, in part, to its ability to form biofilms on biotic surfaces, such as bones and tissues, and abiotic surfaces, such as catheters and other medical devices (10). Biofilms are formed by surface-attached or aggregates of microbial communities that produce a hallmark extracellular polymeric substance that acts as a physical barrier, aiding bacterial survival. Biofilms are challenging to remove or penetrate and can protect bacteria from antimicrobial agents, immune defenses, and desiccation. Due to their persistent nature and the ability to shed cells as they mature, biofilms are commonly associated with infections at surgical sites, catheters, and IV insertion points (11). Inhibitory molecules derived from competitors within the microbiome are an evolving avenue for novel therapeutics against bacterial pathogens (12).

Recent studies have highlighted the role of skin commensals in combating infections caused by pathogenic skin bacteria such as *S. aureus*. For example, lugdunin, a bacteriocin produced by *Staphylococcus lugdunensis*, induces the expression of host-derived AMPs, which dissipate the membrane potential of *S. aureus* (11) and, when combined with *S. epidermidis-*conditioned media, can reduce *S. aureus* colonization by amplifying the innate immune response of the skin (13).

Cell-free conditioned media (CFCM) produced by skin commensals may contain molecules with antibiofilm activity. For example, we have shown CFCM from *Cutibacterium acnes*, an abundant member of the skin microbiome, reduced biofilm formation by both *S. lugdunensis* and *S. hominis* (14). Our group also reported that the CFCM obtained from one *S*. *epidermidis* isolate (RF1) could reduce biofilm formation and disrupt established *S. aureus* biofilms, without affecting *S. aureus* planktonic growth (15). In addition, treatment of biofilm-producing *S. aureus* with CFCM reduced the concentration of the antibiotic needed to eliminate the biofilm (15). In this study, we explored the antibiofilm activity of CFCM obtained from a larger number of bacterial skin commensals from different species. Most of the isolates tested displayed some degree of antibiofilm activity against both MRSA as well as methicillin-susceptible *S. aureus* (MSSA) strains, while *S. epidermidis* isolates exhibited the strongest effect. Size fractionation of the CFCM of three *S*. *epidermidis* isolates suggests that different isolates might produce different molecules contributing to this activity. This suggests that skin microbes that produce antibiofilm molecules could provide new therapeutic avenues to treat intractable infections.

## RESULTS

Of the 79 bacterial isolates collected from different skin sites, 77 were identified at a species level by MALDI-TOF (Table S1). All isolates belonged to the *Staphylococcus* and *Bacillus* genera. *S. epidermidis* was the most commonly isolated species (38 of 79). Other species identified included *Staphylococcus capitis* and *Staphylococcus hominis* (eight each); *Bacillus cereus*, *Staphylococcus haemolyticus,* and *Staphylococcus pasteuri* (five each); *Staphylococcus aureus* (4), *Bacillus licheniformis, Bacillus manliponensis,* and *Bacillus thuringiensis* (one each). Of these, 26 strains were further analyzed for the ability to produce molecules capable of inhibiting *S. aureus* biofilm formation.

**Fig 1:**
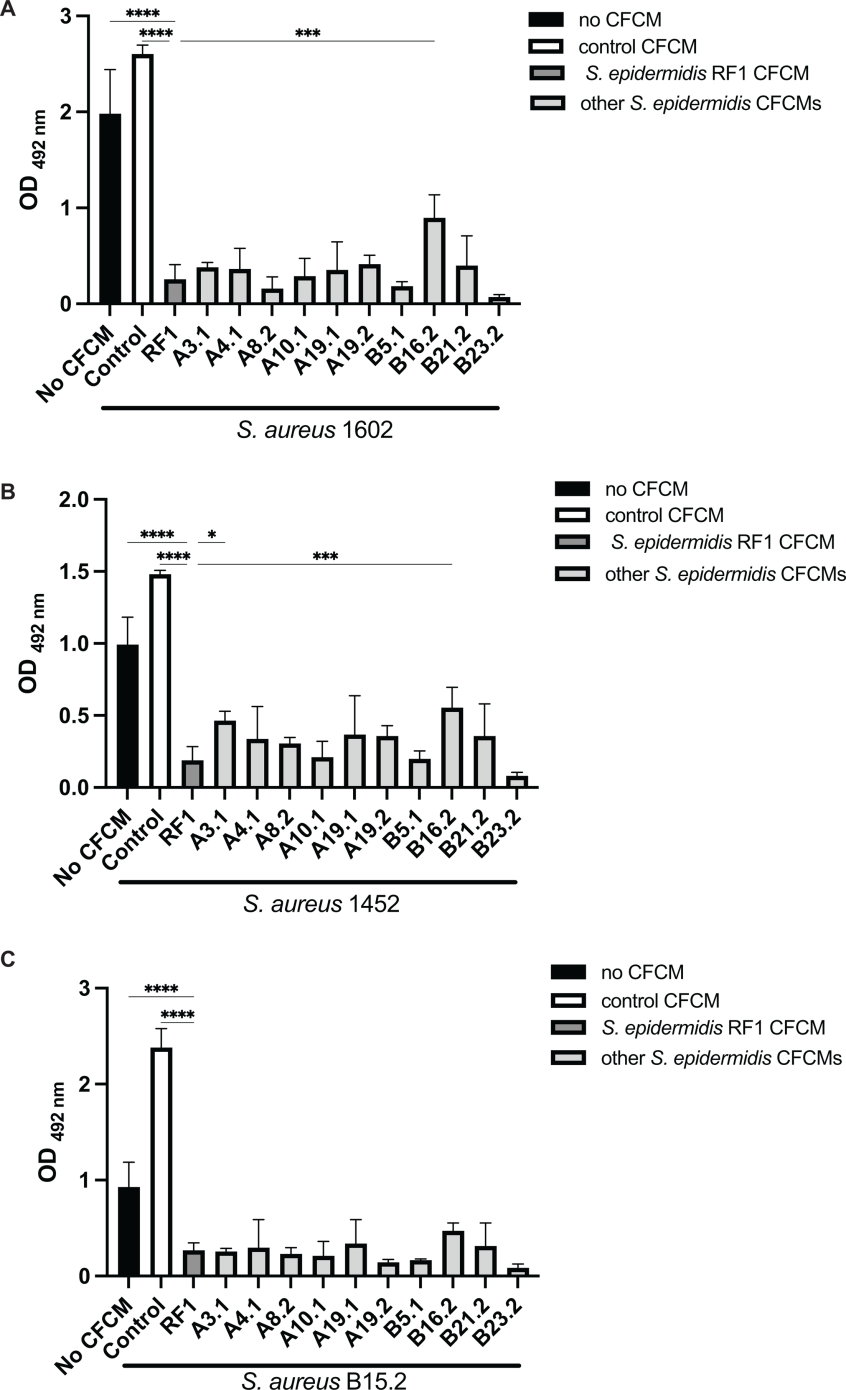
*S. epidermidis* cell-free conditioned media (CFCM) display antibiofilm activity against three different *S. aureus* strains. (**A**) Biofilm formation of *S. aureus* 1602 (MRSA); (**B**) *S. aureus* 1452 (MSSA); (**C**) *S. aureus* B15.2 (MSSA) in the presence or absence of different *S. epidermidis* CFCM. No CFCM: *S. aureus* grown without the addition of CFCM; control: *S. aureus* grown in the presence of concentrated media; RF1: *S. epidermidis* RF1 CFCM. Statistically significant results by one-way ANOVA in comparison with RF1 activity are indicated as *: *P < *0.05, **: *P* < 0.01, ***: *P* < 0.001, and ****: *P* < 0.0001.

CFCM obtained from all *S. epidermidis* strains significantly inhibited biofilm formation on all three *S*. *aureus* strains tested compared to control, albeit to varying degrees (Fig. 1). Compared to the CFCM of *S. epidermidis* RF1, a well-characterized strain with strong anti-biofilm activity (15), the CFCM of B16.2 showed significantly weaker inhibition against two of the three *S*. *aureus* strains (1602 and 1452), indicating reduced anti-biofilm effectiveness, with a 55% average inhibition of *S. aureus* 1602 compared to 87% observed with the CFCM of RF1. Although not statistically significant, the CFCM of one *S*. *epidermidis* isolate, B23.2, had a stronger antibiofilm activity against all *S. aureus* strains compared to RF1, displaying an average inhibition of 96% of the *S. aureus* 1602 biofilm.

**Fig 2:**
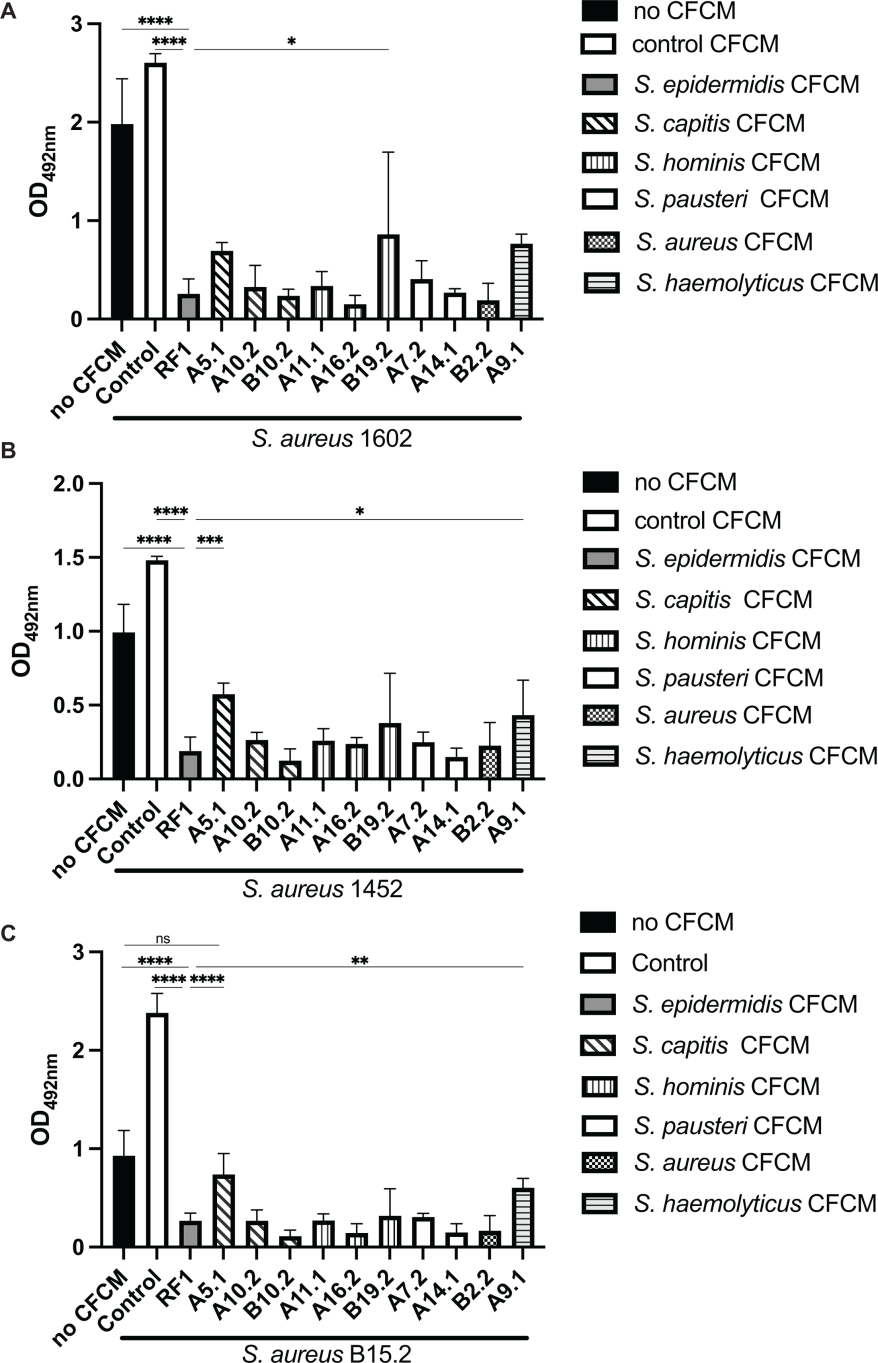
Cell-free conditioned media (CFCM) of non-*epidermidis Staphylococcus* species also display antibiofilm activity against the different *S. aureus* strains. (**A**) Biofilm formation of *S. aureus* 1602 (MRSA); (**B**) *S. aureus* 1452 (MSSA); (**C**) *S. aureus* B15.2 (MSSA) in the presence or absence of different *Staphylococcus* species CFCM. No CFCM: *S. aureus* grown without the addition of CFCM; control: *S. aureus* grown in the presence of concentrated media; RF1: *S. epidermidis* RF1 CFCM. Statistically significant results by one-way ANOVA in comparison with RF1 activity are indicated as *: *P* < 0.05, **: *P* < 0.01, ***: *P* < 0.001, and ****: *P* < 0.0001. Results that were not statistically significant compared to no CFCM are indicated as ns.

CFCM derived from representatives of the other five *Staphylococcus* species isolated also significantly reduced biofilm formation of the three *S. aureus* strains tested, although at different levels (Fig. 2). One exception was the CFCM of *S. capitis* A5.1, which did not significantly inhibit the biofilm formation of *S. aureus* B15.2. In comparison with the activity of *S. epidermidis* RF1, CFCM derived from *S. hominis* B19.2 exhibited a significantly reduced ability to inhibit biofilm formation of *S. aureus* 1602. Furthermore, CFCM derived from *S. capitis* A5.1 and *S. hominis* A9.1 showed a significantly reduced activity against biofilm formation of two *S. aureus* strains, 1452 and B15.2.

**Fig 3:**
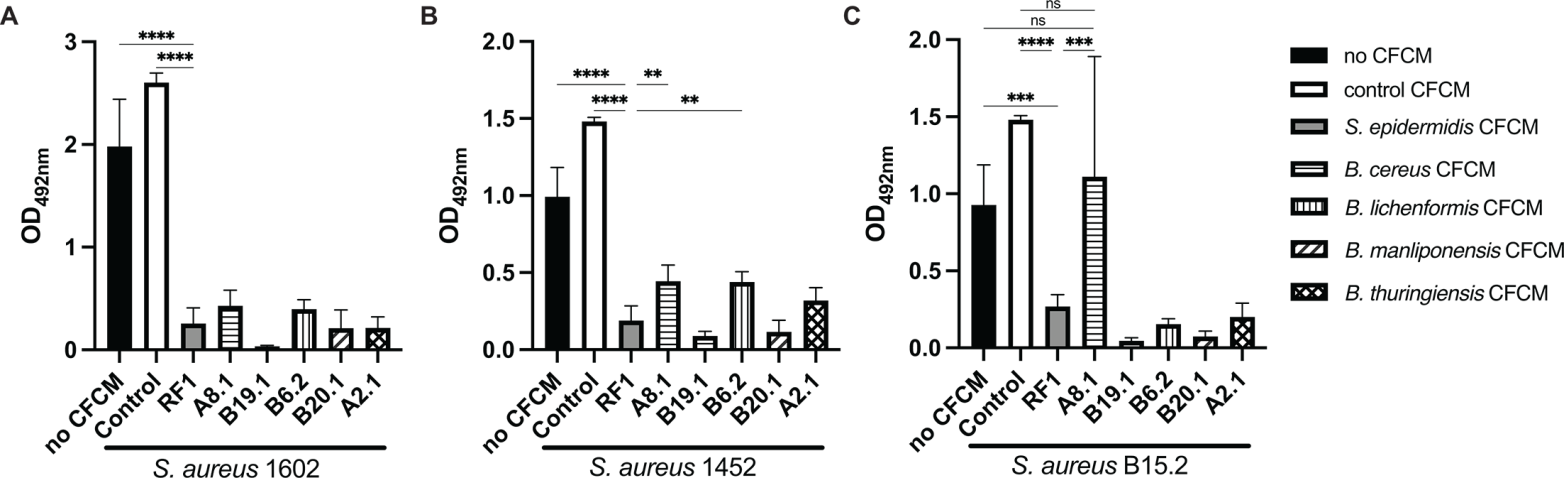
Cell-free conditioned media (CFCM) of *Bacillus* species isolated from the skin impact biofilm formation of three different *S. aureus* strains. (**A**) Biofilm formation of *S. aureus* 1602 (MRSA); (**B**) *S. aureus* 1452 (MSSA); (**C**) *S. aureus* B15.2 (MSSA) in the presence or absence of various *Bacillus* species CFCMs. No CFCM: *S. aureus* grown without the addition of CFCM; control: *S. aureus* grown in the presence of concentrated media. RF1: *S. epidermidis* RF1 CFCM. Statistically significant results by one-way ANOVA in comparison with RF1 activity are indicated as **: *P* < 0.01, ***: *P* < 0.001, and ****: *P* < 0.0001. Results that were not statistically significant compared to no CFCM and control are indicated as ns.

CFCMs generated from the five *Bacillus* strains significantly reduced biofilm formation by *S. aureus* 1602 and 1452 (Fig. 3). However, CFCM derived from *B. cereus* A8.1 did not significantly reduce biofilm formation by *S. aureus* B15.2 and displayed a significantly lower ability to inhibit biofilm formation, as compared to CFCM derived from *S. epidermidis* RF1 on both MSSA strains (1452 and B15.2). Notably, CFCM derived from another *B. cereus* isolate (B19.1) inhibited biofilm formation at higher levels than CFCM derived from *S. epidermidis* RF1 in the three strains tested.

**Fig 4:**
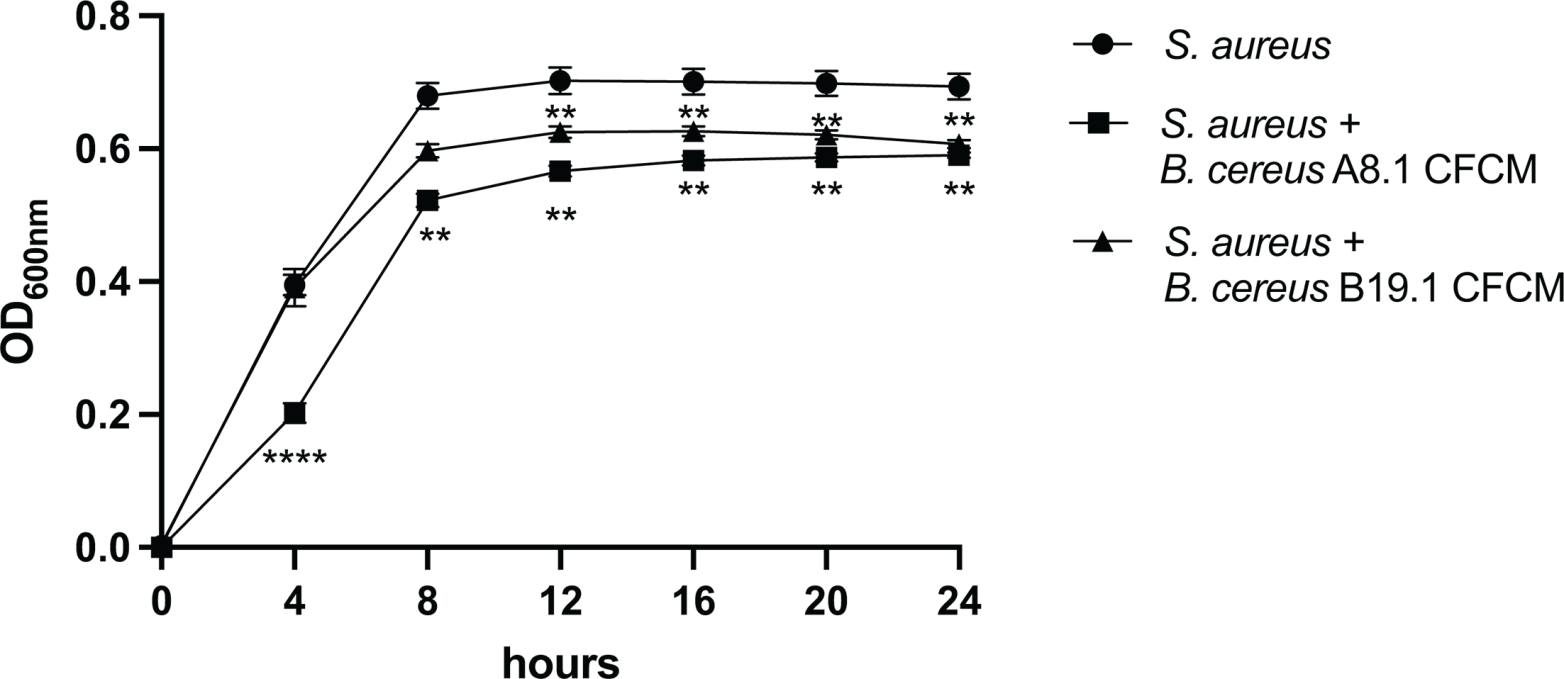
Cell-free conditioned media (CFCM) of two *B. cereus* strains isolated from the skin inhibit planktonic growth of *S. aureus* (1602). **: *P* < 0.01; ****: *P* < 0.0001.

Since we had a significant inhibition of *S. aureus* biofilm formation with most CFCMs, we questioned whether this inhibition could be due to the impact of each CFCM on *S. aureus* planktonic growth. Analysis of the growth curve of *S. aureus* 1602 in the presence and absence of the CFCMs revealed that the CFCMs of most strains did not impact *S. aureu*s planktonic growth (data not shown). The CFCM of the two *B. cereus* isolates (A8.1 and B19.1) had a mild but significant negative impact on *S. aureus* growth (Fig. 4), indicating the effect the CFCM from these isolates displayed on biofilm formation could be due to their inhibitory activity on *S. aureus* replication. However, the average growth inhibition observed when *S. aureus* was grown in the presence of A8.1 CFCM was 14.9% (±0.56%), while biofilm formation was inhibited by 78.3% (±7.63%). In the presence of B19.1 CFCM, *S. aureus* growth was inhibited by 12.5% (±0.87%), while its biofilm formation was reduced by 98% (±0.95%). These observations suggest that the impact of the metabolites produced by the *B. cereus* strains on *S. aureus* biofilm formation cannot be solely explained by planktonic growth inhibition.

**Fig 5:**
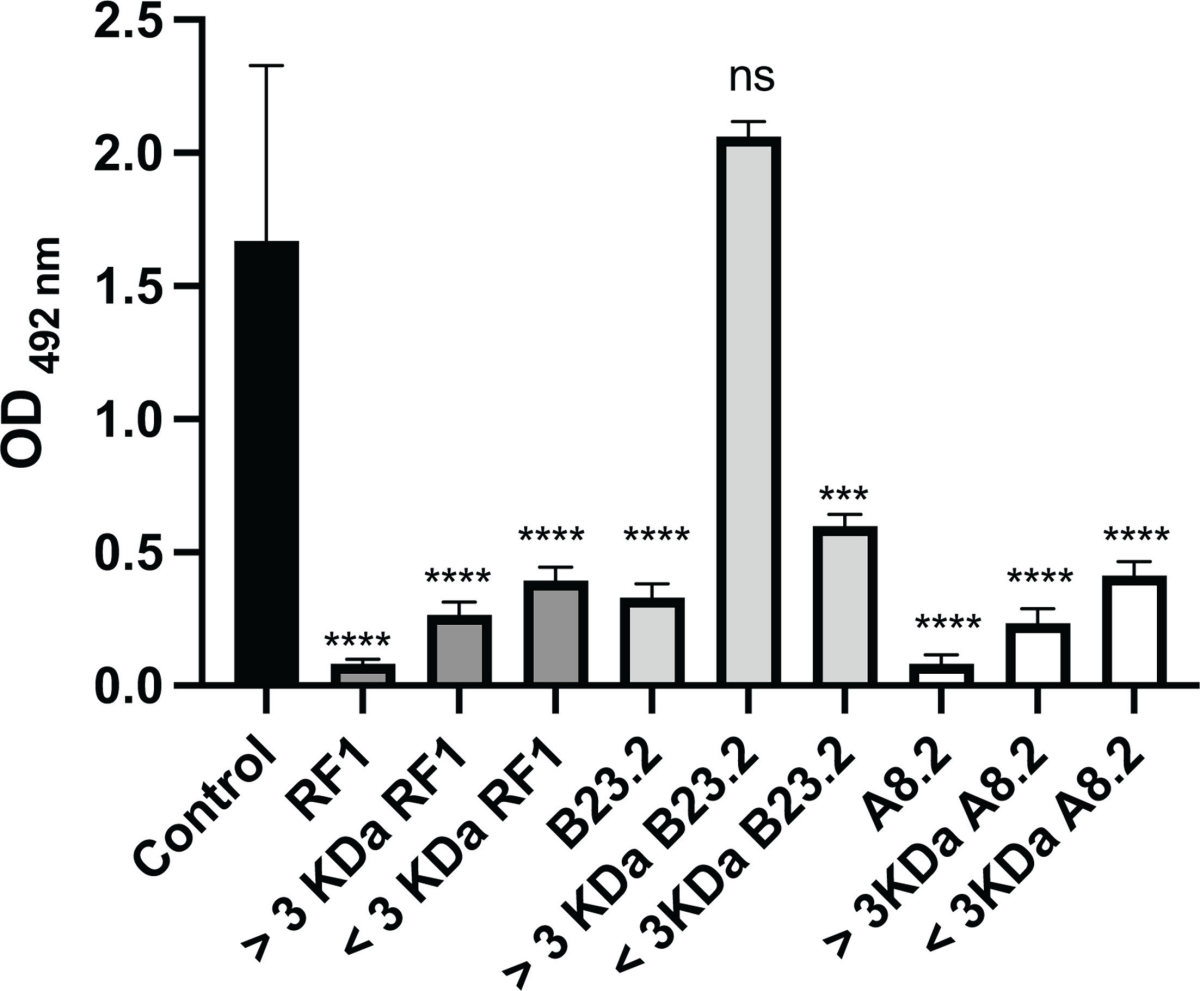
Impact of molecular weight fractionation on the activity of cell-free conditioned media (CFCM) of three *S. epidermidis* strains isolated on biofilm formation of *S. aureus*. Statistically significant results by one-way ANOVA in comparison with *S. aureus* 1602 biofilm formation are indicated as ***: *P* < 0.001, ****: *P* < 0.0001, and ns: not significant.

Size-exclusion fractionation of the metabolites present in three *S. epidermidis* CFCMs with strong antibiofilm activity (RF1, A8.2, and B23.2) showed varying results. Both fractions containing compounds larger (>3 kDa) and smaller than (<3 kDa) 3 kDa from CFCMs obtained from *S. epidermidis* RF1 and A8.2 significantly inhibited biofilm formation by the MRSA strain (1602), although the activity was reduced in comparison with their unfractionated CFCM (Fig. 5). However, only the fraction containing compounds smaller than 3 kDa (<3 kDa) obtained from strain B23.2 retained the ability to inhibit biofilm formation. This suggests strain B23.3 produces a different bioactive compound(s) than strains RF1 and A8.2.

**Fig 6:**
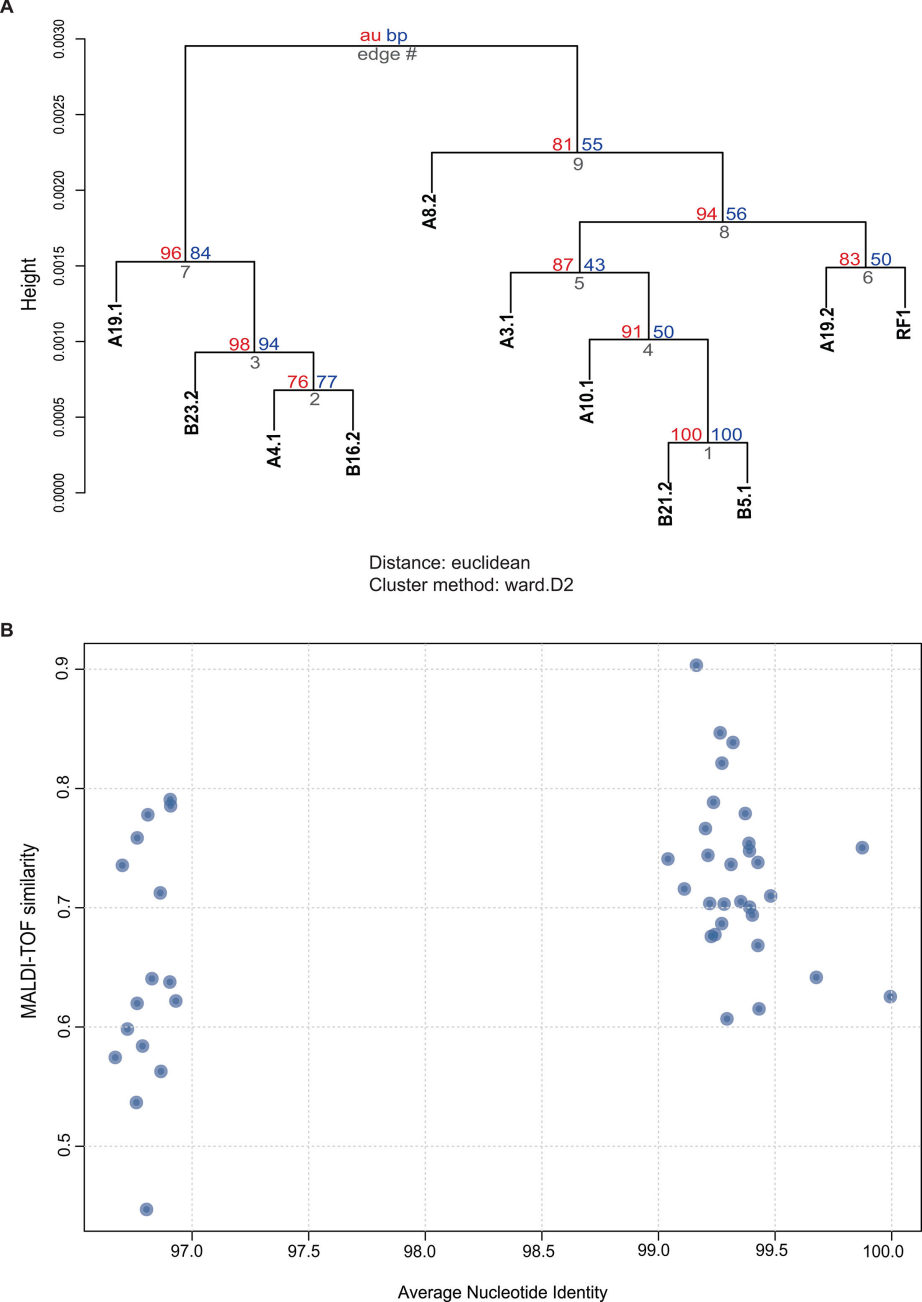
(**A**) The dendrogram obtained by mass spectra cluster analysis with a custom R script represents protein pattern similarities among *S. epidermidis* isolates tested in this study for antibiofilm activity. (**B**) The scatter plot shows the correlation between average nucleotide identity (ANI) and mass spectral similarity (SimCos) for pairwise comparisons among tested *S. epidermidis* isolates.

Comparison of the resolution of MALDI-TOF MS and whole-genome sequencing (Fig. 6) confirmed the high resolution of MALDI-TOF MS for species-level bacterial identification. The similarity between isolates of the same species, assessed by MALDI-TOF MS cosine coefficients, ranged from 0.44 to 0.79, and the ANI ranged from 96.7 to 96.9% (Fig. 6B). These values correlated well with the ANI threshold (>95%) for distinguishing bacterial species. Values for within-strain comparisons ranged from 0.61 to 0.91 for MALDI-TOF MS and from 99 to 100% for ANI. Therefore, MALDI-TOF MS had a moderate resolution when discriminating between strains.

Hierarchical analysis of MALDI-TOF spectra of tested isolates did not reveal any clear relationships between *S. epidermidis* clusters and the ability of these strains to inhibit *S. aureus* biofilm (Fig. 6A). In particular, isolate B23.2, which showed the highest percentage of biofilm inhibition (96%), was included in a cluster (edge number 7) with isolate B16.2, which demonstrated the lowest inhibitory value (55%) among the tested strains. Isolate B16.2, assigned to the subcluster with edge number 2 with isolate A4.1, also differed significantly, 55% and 82%, respectively. The correlation was not detected among isolates from cluster with edge number 9. Thus, B21.2 (the subcluster with edge number 1) had an inhibitory activity of 80% versus 91% for isolate B5.1. Approximately, the same difference was observed between the values for A19.2 and RF1 (subcluster with the edge number 6), 79% and 87%, respectively.

**Fig 7:**
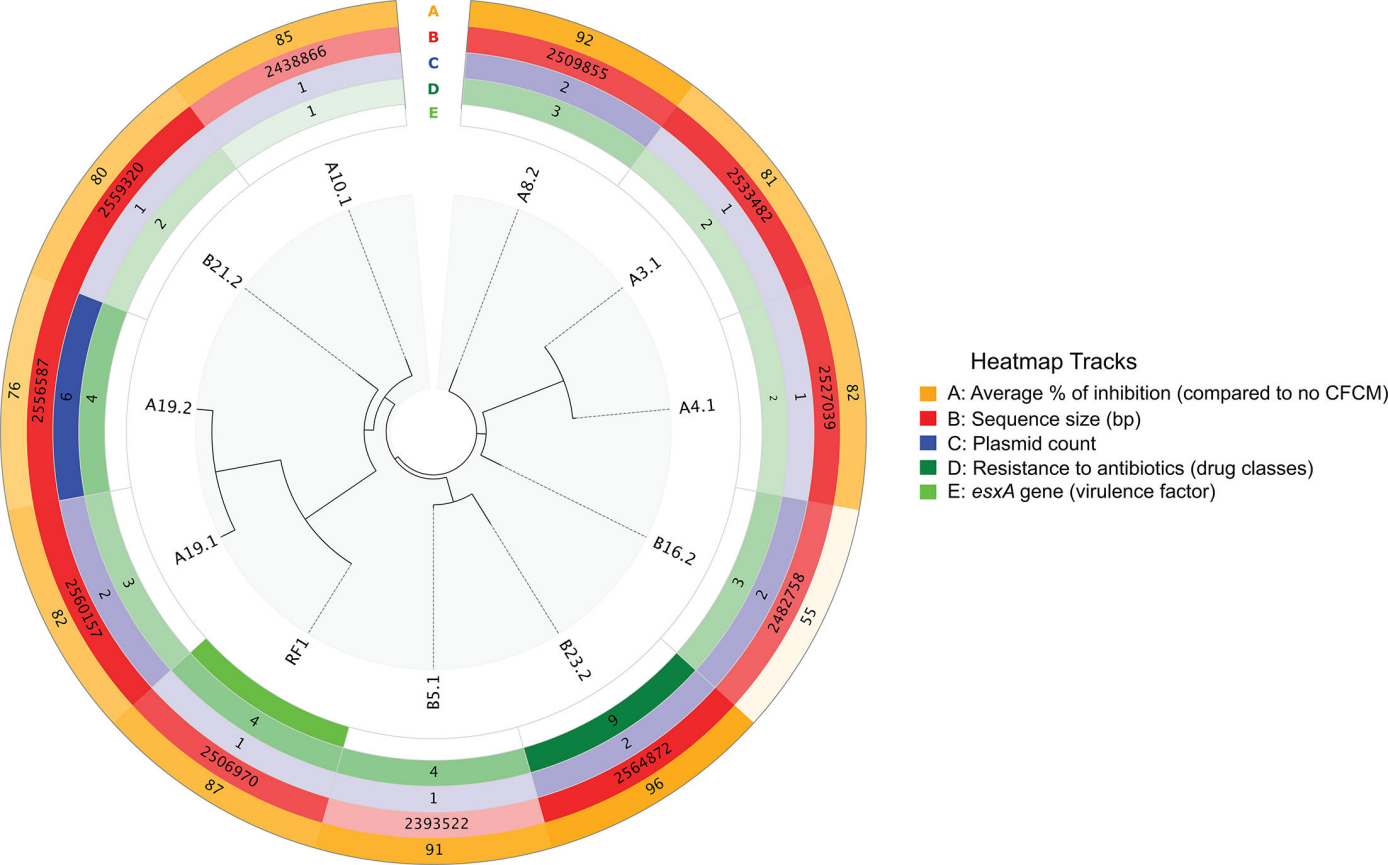
The maximum-likelihood phylogenetic tree based on the core genome alignment of 11 *S. epidermidis* isolates is constructed using Roary and visualized with pyCirclize. Heatmaps represent the information about the average percent biofilm inhibition by isolates (orange), sizes of sequences (red), the number of plasmids (blue), the number of classes of antibiotics to which the isolates are resistant (green), as well as the presence of a virulence factor (lime).

Whole-genome sequencing (WGS) phylogenetic analysis and antibiotic resistance profile of tested *S. epidermidis* isolates (Fig. 7) also did not reveal a direct correlation with their ability to inhibit biofilm formation. Isolates A8.2 and B16.2 were resistant to three classes of antibiotics (Fig. S1) and showed different inhibitive values: 92 and 55%, respectively (Fig. 7). Isolate B23.2 demonstrated the highest antibiotic resistance among the sequenced *S. epidermidis* (nine classes of drugs [Fig. S1]) and had the highest inhibition level (96%). Isolate A10.1, in contrast, was resistant to only one class of antibiotics (Fig. S1) and showed 85% biofilm inhibition, which is around the average inhibition level among the tested isolates (83%). Isolates RF1, A19.1, and A19.2, which were grouped into a clade, in addition to antibiotic resistance, had the *qacC* gene, making them resistant to disinfectants. Altogether, there was no direct correlation between antimicrobial resistance profiles and the ability of these isolates to inhibit *S. aureus* biofilm (Fig. 7).

Given the production of molecules with antibiofilm activity by the different *S. epidermidis* strains, we hypothesized that secondary metabolite biosynthetic gene clusters might be involved in the synthesis of these molecules. Genome sequences were analyzed for the presence of regions with sequence similarities to biosynthetic gene clusters. Most *S. epidermidis* strains tested presented seven biosynthetic gene clusters (Table 1) involved in the production of secondary metabolites: terpene precursors, non-ribosomal peptide synthase, type III polyketide synthases, staphyloferin A, staphylopine, and cyclic lactone autoinducer. Among the two regions for terpene precursors, most strains presented genes encoding for geranylgeranyl pyrophosphate synthase and geranyltransferase. The region for non-ribosomal peptide synthase (NRPS) present in the different strains codified for the thioester reductase domain of alpha aminoadipate reductase Lys2 and NRPSs, while the type III polyketide synthase region codified for hydroxymethylglutaryl-CoA synthase. Two strains (A19.1 and A19.2) presented an additional cluster (terpene), where the main biosynthetic gene codified for squalene synthase; however, its presence did not correlate with different activity than other strains as their average reduction was near the overall average for all the strains (82% and 79%, respectively). One strain (B5.1) had two fewer clusters present (terpene precursor and staphyloferrin A) but still inhibited *S. aureus* biofilm formation by 91%.

**TABLE 1:** Biosynthetic gene clusters present in *S. epidermidis* isolates with inhibitory activity against *S. aureus* biofilm formation

Strain	Average inhibition,^*a*^ % (±SD)	Terpene	Terpene precursor	Cyclic lactone autoinducer	T3PKS	NI-siderophore (staphyloferrin A)	NRPS	Opine-like metallophore (staphylopine)
B23.2	96 (±1.3)	0	2	1	1	1	1	1
A8.2	92 (±6.2)	0	2	1	1	1	1	1
B5.1	91 (±2.4)	0	1	1	1	0	1	1
RF1	87 (±7.7)	0	2	1	1	1	1	1
A10.1	85 (±9.3)	0	2	1	1	1	1	1
A19.1	82 (±14.7)	1	2	1	1	1	1	1
A4.1	82 (±10.8)	0	2	1	1	1	1	1
A3.1	81 (±2.5)	0	2	1	1	1	1	1
B21.2	80 (±15.7)	0	2	1	1	1	1	1
A19.2	79 (±4.6)	1	2	1	1	1	1	1
B16.2	55 (±12.2)	0	2	1	1	1	1	1

^*a*^ Inhibition of *S. aureus* 1602 biofilm formation; SD: standard deviation; T3PKS: type III polyketide synthases, NI-siderophore: non-ribosomal peptide synthetase-independent, IucA/IucC-like siderophores, NRPS: non-ribosomal peptide synthetase. Predictions were made using antiSMASH software version 7.0.

## DISCUSSION

Despite the largely dry, acidic, and salt-coated environment, the skin is home to a diverse microbiome. This community plays a critical role in maintaining the health of the host (16) and, as shown herein, is a hotspot for drug discovery. Specifically, most of the 26 bacterial strains isolated from the skin microbiome of healthy volunteers inhibited the ability of *S. aureus*, an important pathogen, to form biofilms without causing a significant impact on its planktonic growth.

Of the 26 bacteria isolated, 87% were identified as *Staphylococcus* species, which are commonly associated with the skin microbiome, and 11% were identified as *Bacillus* species, which are often regarded as transient commensals, typically associated with soil or environmental sources yet can also be present on the skin of healthy individuals (16, 17). Of the total number of isolates, 48% were *S. epidermidis*, which is frequently isolated from the skin (17). The limited species representation observed in this study is likely attributed to the culture-based methods employed and the inherent bias of culture-based techniques, which tend to overestimate the prevalence of the *Staphylococcus* genus in the skin microbiome (16). In particular, the nutrient-rich media and aerobic incubation conditions used herein are not conducive to isolating species that are abundant in the skin microbiome but require anaerobic growth, such as *C. acnes,* or are fastidious, such as species of *Corynebacterium*. Additionally, bacterial species frequently present in the skin microbiome, including *Micrococcus* and *Streptococcus,* were also not isolated in this study. Furthermore, given the growth conditions used, fungal species that colonize the skin were also not isolated. Consequently, this study does not comprehensively represent the functional diversity of the skin microbiota.

Molecules present in the CFCM of most bacterial isolates tested in this study demonstrated the ability to inhibit biofilm formation by three strains of *S. aureus*. Two of these strains were clinical isolates (MRSA or MSSA), while the third strain was a commensal (MSSA) isolated from the skin of a healthy volunteer as part of this study. Small molecules produced by commensal skin bacteria can inhibit production of biofilms by *S. aureus*. For example, exopolysaccharides derived from *Deinococcus radiodurans,* which can be found in healthy human skin, can inhibit *S. aureus* biofilm formation in a dose-dependent manner (18). Moreover, *D. radiodurans*-produced exopolysaccharide affects different stages of biofilm production, including mature biofilms, by downregulating staphylococcal biofilm-associated genes (*ica* operon). Lugdunin, produced by some strains of *S. lugdunensis*, and its analogs disperse mature *S. aureus* biofilms (19)*.* Additionally, some *S. epidermidis* strains isolated from the nasal cavity secrete a serine protease (Esp) that can inhibit biofilm formation and nasal colonization by *S. aureus* (20). Our group has previously shown that *S. epidermidis* isolated from the skin (strain RF1) secretes molecules other than Esp that can inhibit *S. aureus* biofilm formation, disrupt mature biofilms, and regulate the expression of several virulence genes (15). In this study, our findings demonstrate that metabolites derived from other species isolated from the skin microbiome significantly inhibit *S. aureus* biofilm formation, warranting further investigation of these commensal-derived compounds as novel antibiofilm agents. Furthermore, most CFCMs did not significantly affect *S. aureus* planktonic growth, indicating that their activity was specific to biofilm production. While unveiling the precise mechanisms behind their activity will be the focus of future studies, we anticipate they act on factors critical for biofilm production, rather than bacterial replication. Such factors may include the expression of adhesins, extracellular matrix production, or inducing the dispersion of bacterial cell aggregates. Nevertheless, our work broadens the current knowledge by evaluating a diverse array of strains, including species of *Staphylococcus* and *Bacillus.*

Our data suggest that at least one *S. epidermidis* strain (B23.2) might produce molecules different from the ones produced by strains RF1 and A8.2. Previous characterization of the RF1 bioactive molecule indicated that the molecule is resistant to heat, proteinase K, sodium periodate, as well as protease inhibition treatment. Also, after extraction with ethyl acetate, it fractionates between 3 and 10 kDa (15). In this study, we fractionated the supernatants by molecular weight and for CFCM derived from RF1 and A8.2, fractions smaller and larger than 3 kDa retained antibiofilm activity. This may be due to the presence of multiple bioactive molecules in these supernatants. Alternatively, another hypothesis is that saturation of the filter could have prevented all the bioactive molecules from passing through the filter, leading to molecules smaller than 3 kDa being retained in the larger than 3 kDa fraction. Further chemical characterization, through methods that include high-performance liquid chromatography (HPLC) combined with mass spectrometry, is needed to determine if one or more bioactive molecules are present in these CFCM. Nevertheless, the same phenotype was not observed with isolate B23.2, where only the fraction smaller than 3 kDa inhibited *S. aureus* biofilm formation.

MALDI-TOF is an increasingly common tool for identification of clinically relevant bacterial strains at the species level (21). The utilization of this technology for strain differentiation is evolving, and several groups have evaluated the efficacy of MALDI-TOF for this purpose across various species. For *S. epidermidis* isolates, MALDI-TOF can differentiate biofilm-producing strains versus non-biofilm-producing ones (22), while results in *S. aureus* are more varied. One study boasted a 93% accuracy rate compared with other strain typing methods, but these results had poor reproducibility (23). There is varied success when it comes to strain differentiation in other genera. For example, using the direct transfer method, one group showed 99% strain accuracy of *Escherichia coli* isolates compared with whole-genome sequencing (24). Another group has shown high genetic relatedness of *Bacillus subtilis* species members, which makes strain differentiation by mass spectra difficult (25). Similar studies separately evaluated the efficacy of MALDI-TOF for strain differentiation of *Klebsiella pneumoniae*, *Serratia marcescens*, and *Enterococcus faecium* (21). Here, we report MALDI-TOF MS identified several strains of *S. epidermidis*, as defined by hierarchical clustering (Fig. 6); however, these clusters (as well as clusters determined by WGS analysis) did not clearly correspond to the ability of strains to inhibit biofilm formation. Additionally, cluster alignment from MALDI-TOF did not fully align with those established by WGS, indicating that while MALDI-TOF is a useful tool in strain identification, it is unable to distinguish phylogenetic relationships of *S. epidermidis* as well as WGS analysis.

Biosynthetic gene clusters (BGCs) were detected in all *S. epidermidis* isolates, including some with known function, like staphyloferin A, staphylopine, and cyclic lactone autoinducer. Staphyloferrin A is involved in iron acquisition in *Staphylococcus* spp., while staphylopine is required for metal-sequestering and acquisition, such as Zn^2+^, Ni^2+^, and Co^2+^, in *S. aureus,* and cyclic lactone autoinducer BGC is involved in the synthesis of autoinducer peptides required for signaling of the quorum sensing system in *Staphylococcus* spp. (26) (27, 28). Other BGCs found in the isolates include terpene, terpene precursors, non-ribosomal peptide synthase, and type III polyketide synthases. Notably, the terpene BGC detected in two *S. epidermidis* strains codified for squalene synthesis. Although squalene has been shown to have anti-biofilm activity against *S. aureus* (28), this region was present in two of the *S. epidermidis* strains tested and was absent on the remaining strains that also showed inhibitory activity against the *S. aureus* biofilm. This indicates that although the antibiofilm activity of these two strains may result from squalene production, this is not the case of the other *S. epidermidis* strains.

We have shown that the ability to inhibit *S. aureus* biofilm is a common phenomenon among the members of the skin microbiome. Future work will focus on determining if these skin commensals produce a similar antibiofilm molecule. Developing a broad profile with the characteristics of the bioactive molecules is a good first step in structure determination. Simultaneously, testing the activity of the CFCMs after different treatments, such as sodium periodate, proteases, DNase, and heat, might provide critical information to identify the molecules, as well as determine if the antibiofilm molecules from the different isolates are the same or unique. Active molecules present in the CFCM with similar characteristics could be further purified by, for example, extraction with different solvents and/or high-performance chromatography. Pure active fractions could be analyzed by liquid chromatography coupled with mass spectrometry to determine the identity of these molecules. Furthermore, determining the mechanism of activity of these molecules and their activity against other skin pathogens will help establish their relevance and potential as a therapeutic compound.

There is an unmet need for antimicrobial molecules that are effective against *S. aureus*, given the increasing rates of death associated with antimicrobial resistance among clinical isolates (29). With its constant exposure to the environment and consequently to pathogens, the skin microbiome has the potential to produce molecules that affect pathogen colonization, such as *S. aureus*. Here, we showed that several isolates from different species have the potential to produce molecules that are effective against biofilm production, which is an important factor for pathogen colonization, persistence, and resistance to killing. These molecules could be clinically impactful when used in addition to an antimicrobial, potentiating its activity or rescuing an antibiotic that was no longer effective. Our findings build on current research and contribute to the growing evidence that many microbiome members can shape the behavior of other bacteria within this ecosystem.

## MATERIALS AND METHODS

### Bacterial strains and culture conditions

*S. aureus* strains 1602 and 1452 and *S. epidermidis* strain RF1 were previously isolated from blood infections and identified and characterized by Glatthardt and colleagues (15). The *S. aureus* strains (1602 and 1452) were previously characterized as strong biofilm producers, and *S. epidermidis* strain RF1 was shown to produce and secrete antibiofilm molecules. *S. aureus* strain 1602 is MRSA, whereas strain 1452 is MSSA. All bacterial isolates were stored at ^-^80°C in 20% glycerol stocks and propagated in the lab in tryptic soy agar (TSA) (Thermo Fisher Scientific, Lenexa, KS, USA) and tryptic soy broth (TSB) (Thermo Fisher Scientific, Lenexa, KS, USA) media for 24 h at 37°C. Broth cultures were shaken at 250 rpm.

### Microbiome isolations

Bacterial skin isolates were acquired from 31 student volunteers at the University of Kansas. Samples were collected from exposed skin areas (avoiding regions near the mucosa, palms of the hands, and soles of the feet) using a sterile swab dampened with sterile deionized water, streaked onto either TSA or blood agar plates, and incubated at 37°C for 24–48 h to isolate colonies. Up to two bacterial colonies per student were randomly selected based on the colony morphology and reisolated in TSA or blood agar at 37°C for 24 h to obtain pure cultures. Bacterial isolate identities were confirmed by matrix-assisted laser desorption/ionization time-of-flight mass spectrometry (MALDI-TOF), as described below.

### Bacterial preparation for MALDI-TOF analysis and identification

MALDI-TOF was used for the identification of skin-isolated bacteria using the extended direct protocol (30). Skin commensals were isolated on TSA for 24 h at 37°C. A single large, isolated colony was directly transferred to a polished steel target plate spot with a sterile wooden dowel. The sample was covered with 70% formic acid and allowed to dry at room temperature. After drying, the sample was overlaid with matrix solution (10 mg/mL α-cyano-4-hydroxycinnamic acid [HCCA; Sigma, St. Louis, MO, USA]) in 50% acetonitrile (ACN; Sigma-Aldrich, St. Louis, MO, USA), 47.5% water, and 2.5% trifluoroacetic acid (TFA; Sigma -Aldrich). The bacterial test standard (BTS; Bruker) was prepared according to the manufacturer’s instructions. All isolates were tested in triplicate. For taxonomy analysis, isolates were prepared for MALDI-TOF with the ethanol inactivation and formic acid extraction protocols recommended by Bruker Scientific (Billerica, MA, USA) (31). Briefly, fresh bacterial colonies were transferred with an inoculation loop to a 1.5 mL centrifuge tube with 300 µL LC–MS water (OmniSolv, Sigma-Aldrich) and vortexed. Then, 900 µL of pure ethanol (OmniPur Ethyl Alcohol, Sigma) was added, the tube vortexed, and centrifuged at 15,000 rpm (32,700 × *g*) for 2 minutes. The supernatant was removed, and centrifugation was repeated for 1 minute to remove residual ethanol. The tube with the pellet was dried at room temperature (RT) for 15–20 minutes. The dry pellet was then dissolved in 50 µL of 70% formic acid (Sigma-Aldrich, Steinheim, Germany) and incubated at RT for 5 minutes. Next, 50 µL of acetonitrile (Sigma-Aldrich) was added, and the tubes were thoroughly vortexed. The solution was centrifuged at 15,000 rpm for 2 minutes, and the supernatant was transferred to clean tubes. For MALDI-TOF analysis, 1 µL of the supernatant was spotted on a polished steel target plate. Two spots were prepared for each isolate for identification, and eight spots were used for taxonomic analysis. BTS was placed on two spots, and an additional two spots, which did not contain any isolates, served as negative controls.

### MALDI-TOF spectrophotometry

Mass spectra were acquired on a positive polarity MALDI Biotyper mass spectrometer (Bruker Daltonics, Germany) using the MBT_autoX method with the following parameters: mass range was between 2,000 and 21,000 Da (spectrum size: 21,330 ppt); detector gain: linear, 2,533V; pulsed ion extraction: 390 ns; ion source 1: 19.84 kV; ion source 2: 18.13 kV; lens: 5.96 kV. Smartbeam parameter was in the default mode; the laser frequency: 200 Hz. Real-time smoothing was off, baseline offset adjustment 0%, analog offset −0.7 mV. Each spot was measured with resolution of 0.5 GS/s. Mass calibration was performed in the quadratic mode using default calibration proteins with reference mass from 3,637.8 Da to 16,952.3 Da. The peak assignment tolerance was 1,000 ppm. Obtained mass spectra were identified using the default MALDI Biotyper Library (v11.0.0.0).

### Mass spectra analysis using custom R script

Mass spectra were analyzed as described previously (32). Briefly, a custom script (File S01) was written in R (v4.4.1). This script included functions from packages MALDIquant (v1.22.3) and MALDIquantForeign (v0.14.1) (33), PVclust (v2.2-0 [34]). Mass spectra were aligned twice in loops that tried different values for parameters like half-window size for smoothing intensity and signal-to-noise ratio (SNR) for peak detection. Spectra that did not pass the quality control (low SNR and few peaks) were removed after the first loop. Pairwise cosine similarities and Jaccard coefficients (35) for pairs were calculated. The second loop was used to optimize parameters for MALDI-TOF Taxonomic Unit (MTU) calculation and cluster dendrogram creation.

### Preparation of cell-free conditioned media

Among the identified 77 bacterial isolates, 26 were selected for evaluation of their CFCM activity against biofilm formation. Strains were selected in order to represent the species obtained on our skin microbiome isolations (10 out of 38 *S. epidermidis* isolates, 4 out of 8 *S. capitis*, 3 out of 8 *S. hominis*, 2 out of 5 *S. pasteuri,* 2 out of 5 *B. cereus*, 1 out of 5 *S. haemolyticus,* 1 out of 4 *S. aureus*, 1 out of 2 *B. thuringiensis*, 1 out of 1 *B. licheniformis*, and 1 out of 1 *B. manliponensis*). CFCM of each selected bacterial isolate was prepared as previously described (15). Briefly, a single bacterial colony was inoculated in 50 mL of TSB for 24 h at 37°C with agitation (250 rpm). Cultures were centrifuged (3,100 × *g* for 15 min at 15°C), the supernatant was collected and then filter sterilized (0.22 µm PES filter). The sterile spent media was evaporated to dryness at 45°C using a Speed Vac concentrator (Thermo Fisher Scientific Savant) and resuspended in sterile saline (0.85% NaCl) to 20× its original concentration. The same procedures were performed with culture media (TSB) without the bacterial inoculum and used as a control for the biofilm experiments. To fractionate the CFCM by molecular weight, the unconcentrated cell-free supernatant obtained from *S. epidermidis* isolates (RF1, B23.2, and A8.2) was fractionated using protein concentrators with filters for 3 KDa (Pierce Protein Concentrators, PES For 3K MWCO, Merck Millipore, MA, USA) by centrifugation (swinging bucket, 4,080 × *g* for 75 min). The fractions retained by the filter were called >3 kDa, and the flow through were called <3 kDa. Before testing for activity, fractions 3 kDa were dried using the Speed Vac concentrator and resuspended as described above. Prior to testing, both the >3 kDa and <3 kDa fractions were standardized to 20× concentration. Experiments were performed using three biological replicates and repeated at least two times.

### Biofilm inhibition assay

The activity of CFCM of each selected bacterial isolate was evaluated on *S. aureus* biofilm formation (strains 1602, 1452, and B15.2) using the microtiter plate test, as previously described (15). Briefly, the *S. aureus* inoculum was prepared by adding bacterial colonies in sterile distilled water until reaching an optical density at 600  nm (OD_600_) of 0.1. Then, 120  µL of TSB 1% glucose was supplemented with 15  µL of CFCM, or control CFCM, and 15  µL of the *S. aureus* inoculum was added. Additionally, *S. aureus* biofilm formation was also assayed without any additions. After incubation for 24 h at 37°C, the content of each well was removed, and the wells were carefully washed three times with 200  µL of PBS (pH 7.2) (Thermo Fisher Scientific). The plates were then incubated at 60°C for 1 h and stained with 150  µL per well of 0.1% safranin for 15  min at room temperature. Excess stain was removed by rinsing the wells twice with PBS. The dye was then solubilized using 150  µL of a 95% ethanol solution for 30 min at room temperature, and the optical density at 492  nm (OD_492_) was measured with a microplate reader (SpectraMax, Molecular Devices, San Jose, USA). Results were obtained by subtracting the average ODs of the negative controls (uncultured media) from the average ODs of the experimental wells. Experiments were performed using three biological replicates.

### Bacterial planktonic growth

Growth curves of *S. aureus* 1602 in the presence of or absence of CFCMs were performed on 96-well plates in triplicate. Overnight growth on TSB was diluted to an OD_600_ of 0.05 in TSB with or without the addition of 10% of each CFCM. The microplate was incubated with agitation at 37°C, and the OD_600_ was recorded every hour with a microplate reader (SpectraMax, Molecular Devices). Experiments were performed using three biological replicates.

### Whole-genome sequencing and assembly

The whole-genome sequencing and assembly were performed by Plasmidsaurus using Oxford Nanopore Technology with custom analysis and annotation. The bottom 5% worst fastq reads were excluded using Filtlong (v0.2.1, https://github.com/rrwick/Filtlong, default parameters), and a draft assembly was performed using Miniasm (v0.3) (36). Filtlong (v0.2.1, command line: filtong—mean_q_weight 10) was applied to reduce the read size to ~100× coverage and exclude low-quality reads. Further assembly was performed using Flye (v2.9.1) (37) and Medaka (v1.8.0, https://github.com/nanoporetech/medaka). Analysis of the assembled genome included gene annotation (Bakta, v1.6.1) (38), contig analysis (Bandage, v0.8.1) (39), assessment of assembly completeness and contamination level (CheckM, v1.2.2) (40), and plasmid identification (Mash, v2.3; Sourmash, v4.6.1) (41).

### Phylogenetic analysis

The average nucleotide identity of the whole genomes of the tested isolates was determined using FastANI (v.1.33) (42). Phylogenetic analysis included four genome assemblies of *S. epidermidis* selected from the NCBI Assembly database (GCF_011307235.1, GCF_021398345.1, GCF_024204945.1, and GCF_030013985.1). Prokka (1.14.6) (43) was used for genome annotation. The antimicrobial profile of genomes was determined using AMRFinderPlus (v3.12.8, database v. 2024-01-31.1) (44) and Abricate (v1.0.1) through the virulence factor database (https://github.com/tseemann/abricate) (45); MOB-suite (v3.1.9) was used for plasmid reconstruction and annotation (46). Genomes were also analyzed for the presence of biosynthetic gene clusters using the antiSmash 7.0 web service (https://antismash.secondarymetabolites.org/) (47). Clusters were detected in the relaxed mode with the following extra features: KnownClusterBlast, SubClusterBlast, ActiveSiteFinder, RREFinder, and TFBS analysis. Pan-genome analysis was performed and visualized using roary. GToTree (v1.8.4) (48) was used to construct a phylogenomic tree using genome assemblies from the NCBI Assembly database. The phylogenetic tree was visualized using iTol Online (v7) (https://itol.embl.de/) and pyCirсlize (v1.9.0) (https://github.com/moshi4/pyCirclize).

### Statistical analysis

Biofilm assay comparisons were performed using unpaired one-way ANOVA (Prism 10, GraphPad Software, CA, USA). Growth curve comparisons were performed using Student’s *t*-test of individual points in the curve (Prism 10, GraphPad Software). Differences were considered statistically significant when values of *P*  <  0.05 were obtained.

## SUPPLEMENTAL MATERIAL

Supplemental Material spectrum.01306-25-s0001.docx Figure S1 and Table S1.

ASM does not own the copyrights to Supplemental Material that may be linked to, or accessed through, an article. The authors have granted ASM a non-exclusive, world-wide license to publish the Supplemental Material files. Please contact the corresponding author directly for reuse.
