# Supplementary material for: Metabolites derived from bacterial isolates of the human skin microbiome inhibit Staphylococcus aureus biofilm formation
Source: Microbiol Spectr. 2025 Aug 5;13(9):e01306-25. doi: 10.1128/spectrum.01306-25 (PMC12403628; doi:10.1128/spectrum.01306-25)
Supplement: Supplemental Material — Figure S1 and Table S1. [file spectrum.01306-25-s0001.docx]

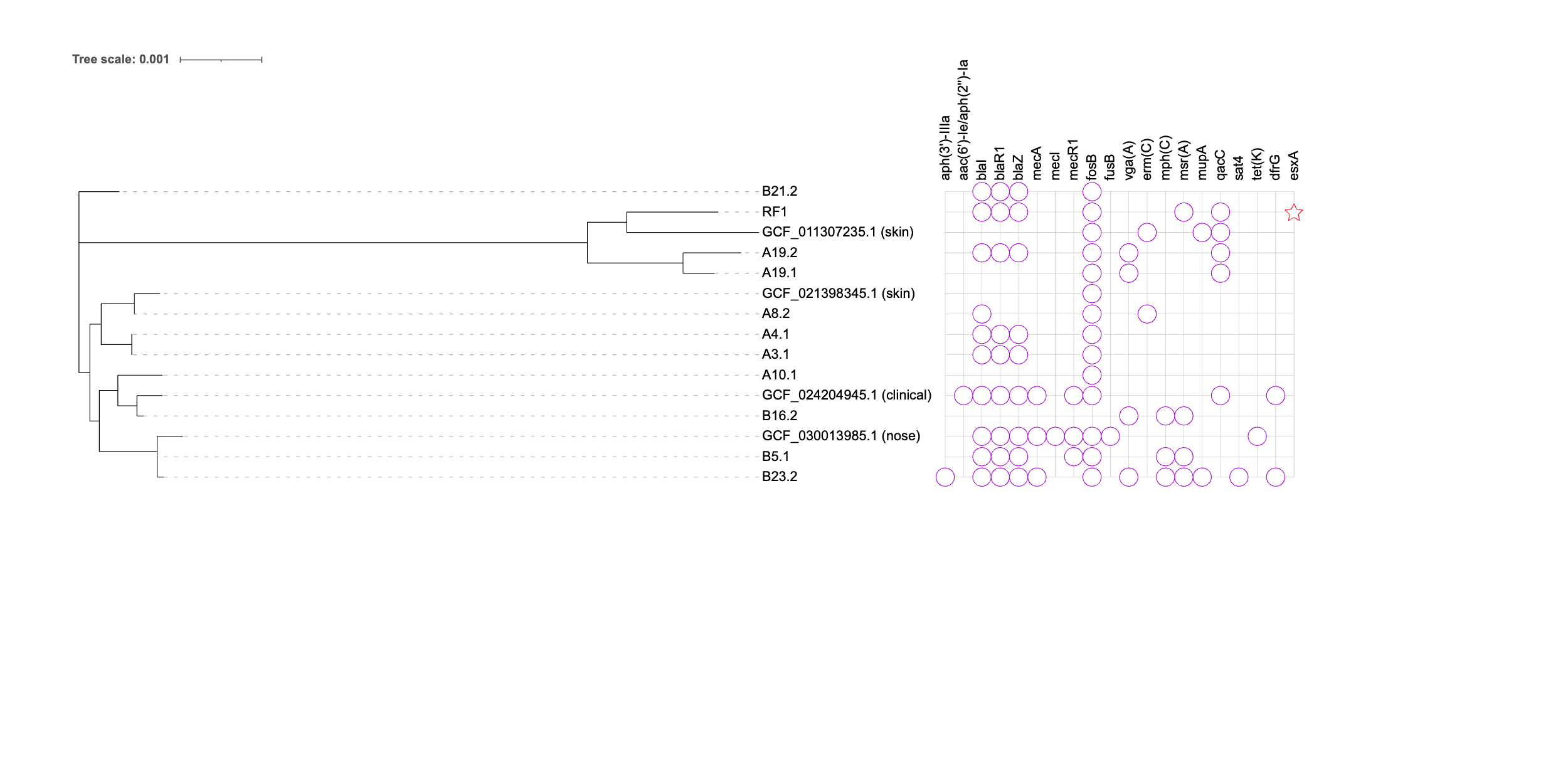


**Supplemental Figure 1:** The phylogenetic tree is constructed using GToTree and supplemented with the antibiotic resistance pattern of the tested isolates. The analysis also includes the relevant genome assemblies of *S. epidermidis* selected from the NCBI Assembly database (GCF_011307235.1, GCF_021398345.1, GCF_024204945.1, GCF_030013985.1). The presence of antibiotic and biocide/disinfectant (*qacC*) resistance genes is marked with purple circles. The red star indicates the presence of *exsA*, a virulence factor secreted via the type VII secretion system (T7SS or ESS). The tree is visualized using iTOL.

Supplemental Table 1. Bacterial species isolated identified from volunteer skin sites in this study.

| **Species identification** | **Number of isolates** | **% of total isolates** | **Isolation skin sites** | **Type of skin sites** |
| --- | --- | --- | --- | --- |
| *Staphylococcus epidermidis* | 38 | 48.1% | Ankle, clavicle, ear, forearm, forehead, inner elbow, leg, neck, scalp, wrist | Sebaceous, moist, dry |
| *Staphylococcus capitis* | 8 | 10.1% | Cheek, forehead, neck, scalp, sternum | Sebaceous |
| *Staphylococcus hominis* | 8 | 10.1% | Ear, forearm, forehead, neck, wrist | Sebaceous, dry |
| *Bacillus cereus* | 5 | 6.3% | Forearm, neck, scalp, wrist | Sebaceous, dry |
| *Staphylococcus haemolyticus* | 5 | 6.3% | Ankle, forearm | Dry, moist |
| *Staphylococcus pasteuri* | 5 | 6.3% | Cheek, ear, forearm, wrist | Sebaceous, dry |
| *Staphylococcus aureus* | 4 | 5.1% | Cheek, neck | Sebaceous |
| *Bacillus lichenformis* | 1 | 1.3% | Forearm | Dry |
| *Bacillus manliponensis* | 1 | 1.3% | Ankle | Moist |
| *Bacillus thuringiensis* | 1 | 1.3% | Knuckle | Dry |
| *Bacillus thuringiensis/mycoides* | 1 | 1.3% | Abdomen | Dry |
| Not identified | 2 | 2.5% | Elbow, forearm | Dry |
